# Systematic Review of Prognostic Role of Blood Cell Ratios in Patients with Gastric Cancer Undergoing Surgery

**DOI:** 10.3390/diagnostics12030593

**Published:** 2022-02-25

**Authors:** Sabine Schiefer, Naita Maren Wirsik, Eva Kalkum, Svenja Elisabeth Seide, Henrik Nienhüser, Beat Müller, Adrian Billeter, Markus W. Büchler, Thomas Schmidt, Pascal Probst

**Affiliations:** 1 Department of General, Visceral and Transplantation Surgery, University of Heidelberg, Im Neuenheimer Feld 420, 69120 Heidelberg, Germany; sabine.schiefer@med.uni-heidelberg.de (S.S.); henrik.nienhueser@med.uni-heidelberg.de (H.N.); beat.mueller@med.uni-heidelberg.de (B.M.); adrian.billeter@med.uni-heidelberg.de (A.B.); markus.buechler@med.uni-heidelberg.de (M.W.B.); pascal.probst@stgag.ch (P.P.); 2 Department of General, Visceral, Cancer and Transplant Surgery, University Hospital Cologne, Kerpener Str. 62, 50937 Köln, Germany; naita.wirsik@uk-koeln.de; 3 The Study Center of the German Society of Surgery (SDGC), University of Heidelberg, Im Neuenheimer Feld 130/3, 69120 Heidelberg, Germany; eva.kalkum@med.uni-heidelberg.de; 4 Institute of Medical Biometry (IMBI), University of Heidelberg, Im Neuenheimer Feld 130/3, 69120 Heidelberg, Germany; seide@imbi.uni-heidelberg.de; 5 Department of Surgery, Cantonal Hospital Thurgau, Pfaffenholzstrasse 4, 8501 Frauenfeld, Switzerland

**Keywords:** gastric cancer, blood cell ratios, prognostic studies, confounder

## Abstract

Various blood cell ratios exist which seem to have an impact on prognosis for resected gastric cancer patients. The aim of this systematic review was to investigate the prognostic role of blood cell ratios in patients with gastric cancer undergoing surgery in a curative attempt. A systematic literature search in MEDLINE (via PubMed), CENTRAL, and Web of Science was performed. Information on survival and cut-off values from all studies investigating any blood cell ratio in resected gastric cancer patients were extracted. Prognostic significance and optimal cut-off values were calculated by meta-analyses and a summary of the receiver operating characteristic. From 2831 articles, 65 studies investigated six different blood cell ratios (prognostic nutritional index (PNI), lymphocyte to monocyte ratio (LMR), systemic immune-inflammation index (SII), monocyte to lymphocyte ratio (MLR), neutrophil to lymphocyte ratio (NLR), and platelet to lymphocyte ratio (PLR)). There was a significant association for the PNI and NLR with overall survival and disease-free survival and for LMR and NLR with 5-year survival. The used cut-off values had high heterogeneity. The available literature is flawed by the use of different cut-off values hampering evidence-based patient treatment and counselling. This article provides optimal cut-off values recommendations for future research.

## 1. Introduction

Gastric cancer (GC) is the fifth most common malignancy, and the third most common cause of cancer-related death worldwide. GC caused 783,000 deaths worldwide in 2018 [1]. While the number of new cases is slowly declining in Europe and the US, Asia continues to record high numbers, and the number of new cases in Africa is increasing. Radical surgery in combination with multimodal therapy remains the only curative treatment option, depending on the tumor stage. Reliable non-invasive diagnostics to further optimize better patient selection or prediction of benefit from surgical therapy or peri-operative therapy are still lacking.

Apart from the tumor cells with defined genetic alterations, which can be targeted in modern treatment regimens [2,3], the tumor communicates/interacts with surrounding cells in the microenvironment. The tumor microenvironment is a very complex process and not completely yet understood. One part of the microenvironment includes inflammatory cells. Virchow first described the presence of leukocytes in neoplastic tissue in 1876 [4]. Until today, numerous studies have shown that systematic inflammation plays a significant role in carcinogenesis and cancer progression [5,6]. The latest publication by Ma et al. presents an overview on the effects of gastric cancer cells on immune cells to induce an immune suppression to protect cancer cells [7]. Understanding the influence of cancer cells on immune reactions may help to find new approaches in cancer treatment.

Over the last few years, inflammatory biomarkers have been investigated in a multitude of studies showing varying prognostic importance.

Systemic inflammation can be measured through blood cell markers, such as lymphocytes, monocytes, neutrophils, and platelets which can be easily and cheaply determined for each patient. Therefore, blood cell ratios and their role as a prognostic factor were intensively investigated in gastric cancer [8,9,10].

The aim of this systematic review was to investigate the prognostic role of blood cell ratios in patients with gastric cancer undergoing surgery in a curative attempt.

## 2. Methods

This systematic review and meta-analysis were carried out in accordance to the PRISMA guidelines [11] and in accordance with recommendations specifically for surgical systematic reviews [12]. The study was conducted according to and registered at PROSPERO 2020 CRD42020164903.

### 2.1. Systematic Literature Search

A systematic literature search was performed in MEDLINE (via PubMed), Web of Science, and Cochrane Central Register of Controlled Trials (CENTRAL) on 4 November 2020 [13]. The following search strategy was performed for MEDLINE:

((gastric*[tiab] OR stomach[tiab]) AND (cancer[tiab] OR carcinoma*[tiab] OR adenocarcinoma*[tiab] OR neoplas*[tiab] OR tumor[tiab] OR tumors[tiab] OR tumour*[tiab] OR malignan*[tiab])) OR “Stomach Neoplasms”[Mesh] OR “GC”[tiab] OR gastrectom*[tiab] OR “Gastrectomy”[Mesh] OR ((gastric*[tiab] OR stomach[tiab]) AND (surgery[tiab] OR surgeries[tiab] OR resection[tiab])) AND “blood ratio”[tiab] OR “platelet count”[tiab] OR “platelet counts”[tiab] OR PLR[tiab] OR (platelet*[tiab] AND lymphocyte*[tiab]) OR SII[tiab] OR “systemic immune inflammation index”[tiab] OR “blood index” [tiab] OR NLR[tiab] OR (neutrophil*[tiab] AND lymphocyte*[tiab]) OR LMR[tiab] OR MLR[tiab] OR (lymphocyte*[tiab] AND monocyte*[tiab]) OR ((CRP*[tiab] OR “C-reactive protein”[tiab]) AND lymphocyte*[tiab]) OR “prognostic nutritional index”[tiab] OR PNI[tiab] OR (albumin*[tiab] AND lymphocyte*[tiab]) OR “inflammation-based prognostic scores”[tiab] OR “preoperative inflammatory parameters”[tiab] OR (“Lymphocyte Count”[Mesh] AND (“Platelet Count”[Mesh] OR “Neutrophils”[Mesh] OR “Monocytes”[Mesh] OR “C-Reactive Protein”[Mesh])) NOT (animals [mh] NOT humans [mh]) NOT “Case Reports” [Publication Type].

The full search strategies for the other databases are available on request.

### 2.2. Study Selection

Prospective and retrospective studies, including patients with gastric adenocarcinoma who underwent curative surgery with or without perioperative chemotherapy, were eligible for inclusion. All studies investigating association of any blood cell ratio (lymphocytes, monocytes, neutrophils, or platelets) with survival were included. Studies on chemotherapy without operation or on palliative surgery were excluded. Animal studies, meeting abstracts, letters, comments, editorials, and publications for which the full text was irretrievable were excluded. There were no restrictions regarding language or publication year.

Titles and abstracts were reviewed independently by two reviewers to select full papers for further evaluation. Any disagreement was resolved by consensus.

### 2.3. Data Extraction

Data were extracted independently by two reviewers using a standardized form. The following items were extracted: Title, first author, country, year of publication, journal, language, study design and period, duration of follow up, sample size, treatment, type of chemotherapy (neoadjuvant and/or adjuvant), cut-off values to define a “high” or “low” ratio, and survival outcomes as overall survival (OS), and disease-free survival (DFS) or as a 5-year survival rate. Furthermore, the area under the curve (AUC), the sensitivity, and the specificity were extracted from the prognostic trials to evaluate discrimination and calibration.

### 2.4. Critical Appraisal (Bias)

For all studies, the risk of bias and quality were assessed by the Quality in Prognosis Studies (QUIPS) tool [14]. Therefore, each of the six domains “participation”, “attrition”, “prognostic factor measurement”, “confounding measurement and account”, “outcome measurement”, and “analysis and reporting” were graded as low risk, moderate risk, or high risk of bias for every study.

Publication bias was explored by funnel plotting, if more than 10 trials were available. In the case of suspected asymmetry, an Egger’s test was performed [15].

### 2.5. Data Handling and Statistical Analysis

Data on cancer-specific survival (CSS) and disease-specific survival (DSS) were considered as OS. Recurrence-free survival, regression-free survival, relapse-free survival, and progression-free survival were considered as DFS. For the 5-year survival rate, the univariate analysis of overall survival was used. If there was a training and validation set, the data of the validation set were extracted. If hazard ratios (HR) were not explicitly reported, the HR was determined by using Tierney’s method [16]. The original survival curves were extracted using WebPlotDigitizer [17] to calculate HR.

Primary statistical analysis and meta-analysis were performed with program R using the extensions meta and diagmeta [18,19,20]. A random-effects model was used to account for methodological and clinical heterogeneity. Statistical heterogeneity among the effect estimates of the included trials was evaluated using the I^2^ statistic. An I^2^ less than 25% was considered to indicate low heterogeneity and an I^2^ > 75% was used to indicate high heterogeneity. The 5-year survival rate was pooled as odds ratio (OR) with a 95%-CI using the Mantel–Haenszel (M-H) method. OS and DFS were pooled as hazard ratios using the method of DerSimonian and Laird.

Optimal cut-off values were investigated in a stepwise approach and recommendations were made according to the strength of the used method. In case of enough homogenous identified studies, the optimal cut-off value for future prognostic studies was defined quantitatively using summary receiver operating characteristic (SROCs) to identify the combined cut-off value with the highest association by the pooled estimate [21]. A high grade of recommendation was given for optimal cut-off values defined by SROC.

Further, meta-regression using the study-specific cut-off values were used to adjust all analyses for varying definitions of “low” vs. “high” marker concentrations if three or more studies contributed data to an analysis. A moderate grade of recommendation was given if a statistically significant association between the varying cut-off values and the outcome was observed.

In case of lack of data in the included studies, a cut-off value for future prognostic studies was searched qualitatively by the lowest/highest cut-off value above which still shows a significant association with overall survival. A low grade of recommendation was given for optimal cut-off values defined by qualitative methods.

Results were graphically illustrated by means of forest plots and SROC visualizations. Results for meta-regressions were tabulated and publication bias was assessed by means of funnel plots.

## 3. Results

**Figure 1:**
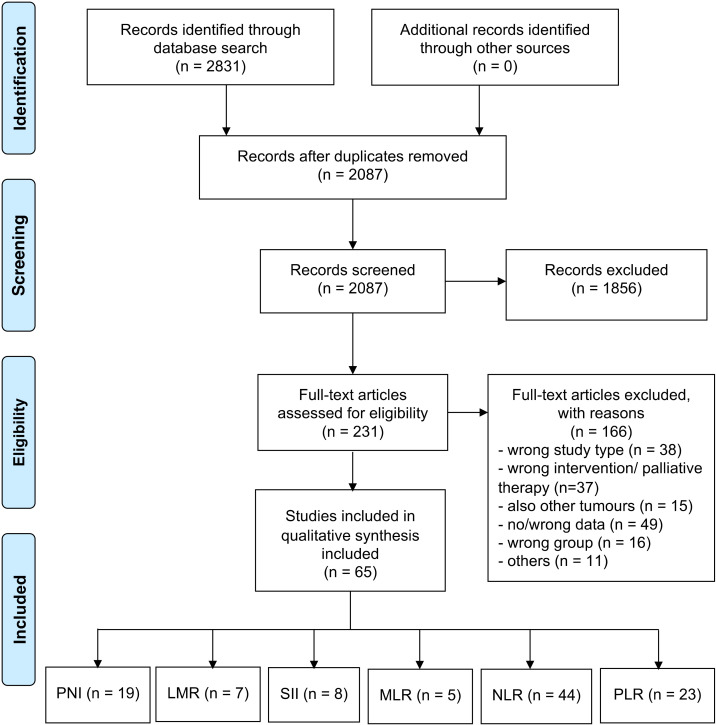
PRISMA flow diagram showing selection of included articles for review. Abbreviation: PNI: Prognostic nutritional index; LMR: Lymphocyte to monocyte ratio; SII: Systemic immune-inflammation index; MLR: Monocyte to lymphocyte ratio; NLR: Neutrophil to lymphocyte ratio; and PLR: Platelet to lymphocyte ratio.

A total of 2831 articles were screened for eligibility. A total of 231 of these trials were assessed in full text. From these, 166 trials were excluded because of incorrect study type (n = 38), incorrect intervention or palliative operation (n = 37), investigation of other tumors (n = 15), incorrect or no published data (n = 49), incorrect cancer stage or population (n = 16), and other reasons (n = 11). Finally, 65 studies were included in the qualitative and quantitative synthesis. A PRISMA flow chart is shown in Figure 1.

In all studies, patients were resected in a curative intent. In 24 studies, the therapy was surgery only, whereas in 39 trials, the patients had additional neoadjuvant or adjuvant chemotherapy. For 2 studies, there was no information available.

Most of the studies excluded patients treated with neoadjuvant chemotherapy, and just 12 studies were enrolled, which used neoadjuvant treatment.

In 7 studies, the blood was taken before surgery, otherwise before neoadjuvant chemotherapy. The variety of the blood measurement was between “day before surgery” up to “2 months before surgery” or “time of diagnosis”. In 34 of the 65 studies, the patient’s blood was taken 1–2 weeks before treatment and just 7 studies took a sample after neoadjuvant chemotherapy. The patients’ number was very low in comparison to the cohort (6x <5% of the patients, 1x 10% and 1x 13.9%). Just 7 studies had no information about the time the blood was taken.

The majority of the published studies were performed in Asia (60 of 65 studies: 92.3%). Four studies were performed in Italy (6.2%) and one in Brazil (1.5%).

The included studies investigated the following blood cell ratios: Prognostic nutritional index (PNI), lymphocyte to monocyte ratio (LMR), systemic immune-inflammation index (SII), monocyte to lymphocyte ratio (MLR), neutrophil to lymphocyte ratio (NLR), and platelet to lymphocyte ratio (PLR) (Table 1).

### 3.1. Qualitative Analysis

For the domain “participation” there was only one study from 65 studies at high risk of bias (1.5%). A total of 29 of 65 studies (44.6%) were at moderate risk of bias and 34 of 65 studies (52.3%) were at low risk of bias. For one from 65 studies (1.5%), the bias remained unclear.

For the domain “attrition”, there were 24 from 65 studies at high risk of bias (36.9%). A total of 37 of 65 studies (56.9%) were at moderate risk of bias and 3 of 65 studies (4.6%) were at low risk of bias. For one from 65 studies (1.6%) the bias remained unclear.

For the domain “prognostic factor measurement”, there were 5 from 65 studies at high risk of bias (7.7%). A total of 56 of 65 studies (86.2%) were at moderate risk of bias and 3 of 65 studies (4.6%) were at low risk of bias. For one from 65 studies (1.5%), the bias was unclear.

For the domain “outcome measurement”, there were 8 from 65 studies at high risk of bias (12.3%). A total of 39 of 65 studies (60.0%) were at moderate risk of bias and 17 of 65 studies (26.2%) were at low risk of bias. For one from 65 studies (1.5%), the bias remained unclear.

For the domain “confounding measurement and account”, there were 25 from 65 studies at high risk of bias (38.5%). A total of 29 of 65 studies (44.6%) were at moderate risk of bias and 9 of 65 studies (13.8%) were at low risk of bias. For two from 65 studies (3.1%), the bias remained unclear.

For the domain “statistical analysis and reporting”, there were 5 from 65 studies at high risk of bias (7.7%). A total of 45 of 65 studies (69.2%) were at moderate risk of bias and 14 of 65 studies (21.6%) were at low risk of bias. For one from 65 studies (1.5%), the bias remained unclear. Table 2 gives an overview of the risk of bias assessment according to QUIPS.

**Figure 2:**
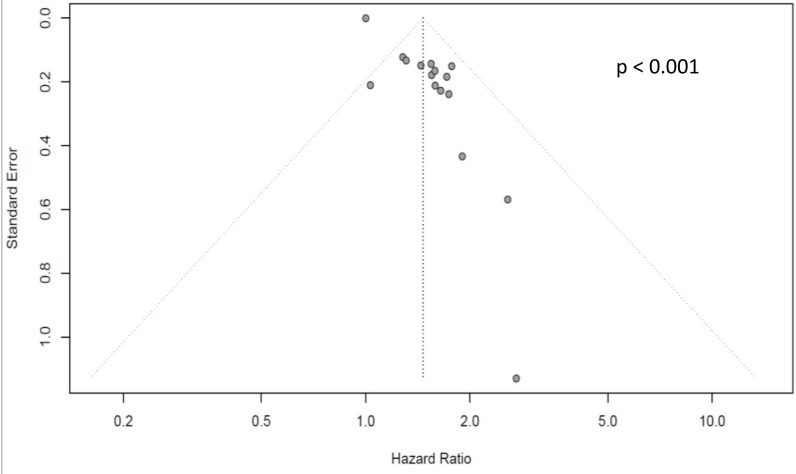
Funnel plot for PLR showing significant asymmetry, indicating a publication bias.

There were enough studies to analyze publication bias for OS of PNI, PLR, and NLR, for DFS of NLR, and for the 5-year survival rate of NLR. There was a statistically significant asymmetry indicating publication bias for OS of PLR (Figure 2, *p* < 0.001). All other funnel plots did not show asymmetry (Supplementary Figure S1A–D, all *p* > 0.05).

### 3.2. Quantitative Analysis

In the studies, different cut-off values were used. The cut-off values of all following analysis are shown in Table 3.

#### 3.2.1. Prognostic Nutritional Index (PNI)

PNI was investigated by 19 studies [22,23,24,25,26,27,28,29,30,31,32,33,34,35,36,37,38,39,40]. A high PNI is hypothesized to be associated with a longer survival.

Performing a SROC analysis was not possible due to high heterogeneity.

**OS**

**Figure 3:**
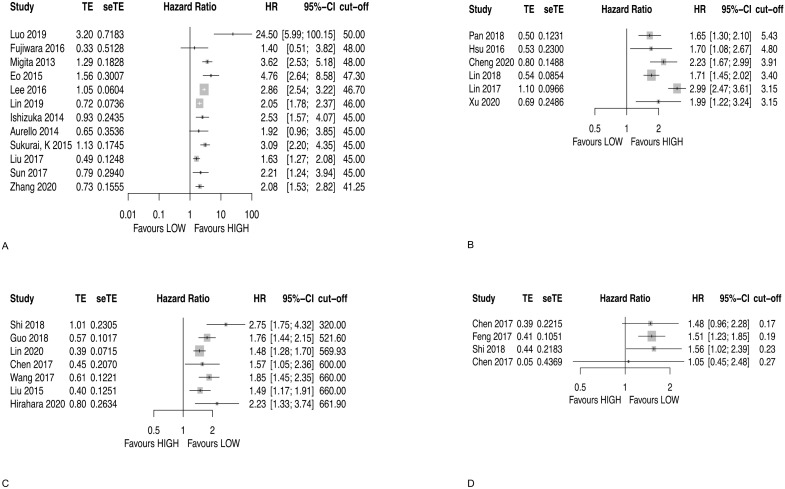
Forest plot for OS for (**A**) PNI, (**B**) LMR, (**C**) SII, and (**D**) MLR.

Twelve studies analyzed PNI for OS [22,23,24,25,26,27,28,29,30,31,32,40]. In 10 studies, a high PNI was significantly associated with a longer OS, whereas in two studies there was no significant association (Figure 3A and Table 3).

The meta-regression showed that the association of a PNI with longer OS was significantly explained by a higher cut-off value (*p* = 0.026) (Supplementary Figure S2A).

**DFS**

Five studies analyzed PNI for DFS [22,23,26,29,40]. All studies showed a significant association of longer DFS and high PNI (Supplementary Figure S3A and Table 3).

The meta-regression indicated that the association of a PNI with a longer DFS was significantly explained by a higher cut-off value (*p* = 0.032) (Supplementary Figure S2B), albeit these results are based upon few studies only.

**5-Year survival rate**

Ten studies analyzed PNI for the 5-year survival rate [23,30,32,33,34,35,36,37,38,39]. All studies showed a significant association of a higher 5-year survival rate and high PNI (Supplementary Figure S3B and Table 3).

The meta-regression showed that the association of a PNI with the 5-year survival rate was not significantly explained by a higher cut-off value (*p* = 0.284) (Supplementary Figure S2C).

#### 3.2.2. Lymphocyte to Monocyte Ratio (LMR)

LMR was investigated by seven studies [41,42,43,44,45,46,47]. A high LMR is hypothesized to be associated with a longer survival.

Performing a SROC analysis was not possible due to lack of data.

**OS**

Six studies analyzed LMR for OS [41,42,43,44,46,47]. All studies were significantly associated with a longer OS and high LMR at the used cut-off values (Figure 3B and Table 3).

The meta-regression showed that the association of an LMR with longer OS was not significantly explained by a higher cut-off value (*p* = 0.253) (Supplementary Figure S2D).

**DFS**

Three studies analyzed LMR for DFS [42,45,47]. All studies showed a significant association of a longer DFS and high LMR at the used cut-off values (Supplementary Figure S3C and Table 3).

The meta-regression showed that the association of an LMR with a longer DFS was not significantly explained by a higher cut-off value (*p* = 0.853) (Supplementary Figure S2E).

**5-Year survival rate**

Three studies analyzed LMR for the 5-year survival rate [41,44,45]. All studies showed a significant association of a higher 5-year survival and high LMR (Supplementary Figure S3D and Table 3).

The meta-regression indicated that the association of an LMR with the 5-year survival rate was significantly explained by a higher cut-off value (*p* = 0.020) (Supplementary Figure S2F), albeit this result is based on few studies only.

#### 3.2.3. Systemic Immune-Inflammation Index (SII)

SII was investigated by eight studies [48,49,50,51,52,53,54,55]. A low SII is hypothesized to be associated with a longer survival.

Performing SROC analysis was not possible due to lack of data.

**OS**

Seven studies analyzed SII for OS [48,49,50,51,52,54,55]. All studies were significantly associated with a longer OS and low SII (Figure 3C and Table 3).

The meta-regression showed that the association of a SII with longer OS was not significantly explained by a lower cut-off value (*p* = 0.171) (Supplementary Figure S4A).

**DFS**

Two studies analyzed SII for DFS [48,53]. Both studies showed a significant association of longer DFS and low SII (Supplementary Figure S3E and Table 3). No meta-regression could be performed due to lack of data.

#### 3.2.4. Monocyte to Lymphocyte Ratio (MLR)

MLR was investigated by five studies [48,51,56,57,58]. A low MLR is hypothesized to be associated with a longer survival.

Performing a SROC analysis was not possible due to lack of data.

**OS**

Four studies analyzed MLR for OS [48,51,56,57]. In two studies, a low MLR was significantly associated with a longer OS, whereas in two studies there was no significant association (Figure 3D and Table 3).

The meta-regression indicated that the association of an MLR with a longer OS was not significantly explained by a lower cut-off value (*p* = 0.695) (Supplementary Figure S4B), albeit this analysis was based on few studies only.

**DFS**

Three studies analyzed MLR for DFS [48,56,58]. One study showed a significant association of longer DFS and low MLR, whereas in two studies there was no significant association (Supplementary Figure S3F and Table 3).

The meta-regression showed that the association of an MLR with a longer DFS was not significantly explained by a lower cut-off value (*p* = 0.660) (Supplementary Figure S4C).

#### 3.2.5. Neutrophil to Lymphocyte Ratio (NLR)

NLR was investigated by 44 studies [22,25,36,40,43,44,45,46,48,49,50,51,52,53,54,57,58,59,60,61,62,63,64,65,66,67,68,69,70,71,72,73,74,75,76,77,78,79,80,81,82,83,84]. A low NLR is hypothesized to be associated with a longer survival.

Performing SROC analysis from 17 studies [36,39,46,50,51,52,53,54,58,61,62,73,74,76,81,83,85] resulted in an optimal cut-off value of 4.506. The sensitivity and specificity at this cut-off value were 0.378 (95%-CI: 0.151 to 0.675) and 0.863 (95%-CI 0.648 to 0.955), respectively (Supplementary Figure S5A).

**OS**

**Figure 4:**
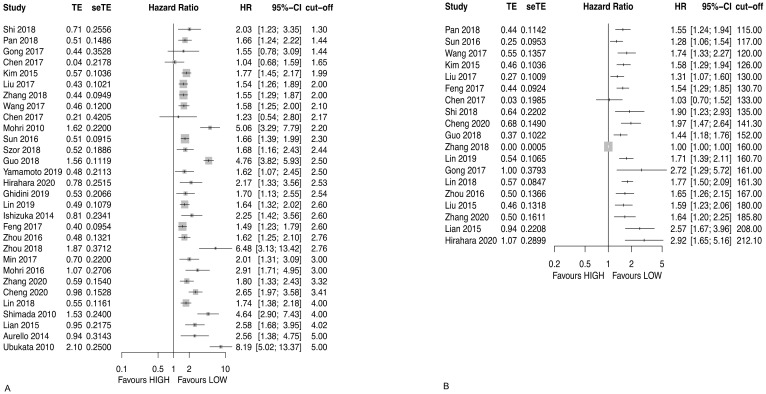
Forest plot for OS for (**A**) NLR and (**B**) PLR.

Thirty studies analyzed NLR for OS [22,25,40,43,44,46,48,49,50,51,52,54,57,59,60,61,62,63,64,65,66,67,68,69,70,71,72,73,74,75]. In 27 studies, a low NLR was significantly associated with a longer OS, whereas in three studies there was no significant association (Figure 4A and Table 3).

The meta-regression showed that the association of an NLR with longer survival was significantly explained by a lower cut-off value (*p* = 0.002) (Supplementary Figure S4D).

**DFS**

Eleven studies analyzed NLR for DFS [22,40,45,48,53,58,63,70,72,74]. In nine studies, a low NLR was significantly associated with a longer DFS and low NLR, whereas in two studies there was no significant association (Supplementary Figure S6A and Table 3).

The meta-regression showed that the association of an NLR with longer survival was significantly explained by a lower cut-off value (*p* = 0.006) (Supplementary Figure S4E).

**5-Year survival rate**

Eighteen studies analyzed NLR for a 5-year survival rate [36,44,45,65,66,68,71,72,73,76,77,78,79,80,81,82,83,84]. Fifteen studies showed a significant association of a higher 5-year survival and low NLR, whereas in three studies there was no significant association (Supplementary Figure S6B and Table 3).

The meta-regression showed that the association of an NLR with the 5-year survival rate was significantly explained by a lower cut-off value (*p* = 0.001) (Supplementary Figure S4F).

#### 3.2.6. Platelet to Lymphocyte Ratio (PLR)

PLR was investigated by 23 studies [40,43,44,46,48,49,50,51,52,53,54,57,61,62,63,64,69,73,75,78,79,85,86]. A low PLR is hypothesized to be associated with a longer survival.

Performing SROC analysis from 11 studies [39,46,51,52,53,54,61,62,73,85,86] resulted in an optimal cut-off value of 152.47. The sensitivity and specificity at this cut-off value were 0.55 (95%-CI: 0.45 to 0.65) and 0.62 (95%-CI 0.53 to 0.70), respectively (Supplementary Figure S5B).

**OS**

Nineteen studies analyzed PLR for OS [40,43,44,46,48,49,50,51,52,54,57,61,62,63,64,69,73,75,85]. In 18 studies, a low PLR was significantly associated with a longer OS, whereas in one study there was no significant association (Figure 4B and Table 3).

The meta-regression showed that the association of a PLR with longer OS was not significantly explained by a lower cut-off value (*p* = 0.144) (Supplementary Figure S7A).

**DFS**

Five studies analyzed PLR for DFS [40,48,53,62,63]. In three studies, a low PLR was significantly associated with a longer DFS, whereas in two studies there was no significant association (Supplementary Figure S6C and Table 3).

The meta-regression showed that the association of a PLR with longer DFS was not significantly explained by a lower cut-off value (*p* = 0.659) (Supplementary Figure S7B).

**5-Year survival rate**

Four studies analyzed PLR for the 5-year survival rate [44,78,79,86]. All studies showed a significant association of a higher 5-year survival and low PLR (Supplementary Figure S6D and Table 3).

The meta-regression showed that the association of a PLR with the 5-year survival rate was not significantly explained by a lower cut-off value (*p* = 0.629) (Supplementary Figure S7C).

Table 3 shows a summary of the quantitative results.

### 3.3. Blood Cell Ratio Recommendation

To form a basis for future research on blood cell ratios as a biomarker, optimal cut-off values were calculated and suggested for future use (Table 4).

While for PNI a validated cut-off value exists of 45 [87], which is recommended for future use, only a minority of the included studies used this validated cut-off value. Interestingly, one of the studies also did not show a significant result when using this cut-off value.

For LMR, it was not possible to perform SROC analysis; the highest cut-off value for a significant association was 5.43, which is a suggestion for further research.

For SII, the highest cut-off value for a significant association was 320, therefore it is recommended to use for further research as SROC analysis was not possible to perform.

For MLR, the cut-off value recommendation using the results of this study is 0.19 even when SROC analysis was not possible because of leaking data.

For NLR, the optimal cut-off value found by SROC analysis was 4.506, therefore the cut-off value recommendation for further research of NLR is 4.5.

For PLR, the optimal cut-off value found by SROC analysis was 152.47, therefore the cut-off value recommendation for further research of PLR is 152.

## 4. Discussion

The aim of this systematic review was to investigate the prognostic role of blood cell ratios in patients with gastric cancer undergoing surgery in a curative attempt. More than 60 included studies investigated blood cell ratios for their impact on gastric cancer prognosis. This large body of evidence in the current literature highlights the relevance and importance of this topic, as simple and strong biomarkers for prognosis are warranted to better advise patients and to also develop new and adapted treatment strategies for patients with a worse prognosis.

In recent years, several blood cell ratios have been identified and evaluated as prognostic markers for patients with gastric cancer. While such prognostic markers (and their potential cut-off values) are needed for oncological management, several methodological specifics are to be considered so as to make a valid statement when identifying and evaluating them. Unfortunately, not all of the studies identified in this review follow the necessary statistical principles with enough rigor. A clinically valuable prediction model needs to distinguish between development, validation, and an implementation step [88,89,90]. In the development stage, prospective planning of the study and the definition of the study population are important to obtain generalizability in the targeted population [91]. A clinically meaningful prediction model needs to predict probabilities that are in line with the actual outcome frequencies in observed patients (calibration) and needs to be able to distinguish between diseased and non-diseased patients (discrimination). Therefore, both should be assessed and reported along with the model itself. In the identified studies, measures of calibration and discrimination are often missing, and the respective studies could not be used in the prognostic meta-analytic model. Another commonly known problem in the development of predictive markers is the fact that the models are fitted optimally to the observed sample, which results in an overoptimistic assessment of calibration and discrimination (also known as overfit) [92]. Strategies to avoid overfitting include different methods for internal validation [92], such as cross validation or bootstrapping, which are based on randomly sampling from the observed data to obtain training and validation set(s). Besides internal validation, external validation on an independent patient cohort is essential, as it is not guaranteed that a good model fits equally well to a different cohort [93,94]. The final step, the implementation in practice, regards the quantification of the impact in clinical care and the adaption to medical practice [94]. This implies that published, well-established predictive marker(s) (and their potential cutoffs) can be used in a subsequent trial. While improving existing prediction models can be benchmarked against well-established ones, using the median of an observed patient cohort to define a new cut-off value (a data-driven approach dependent on the sample at hand) will not, in general, lead to valid and generalizable results [89].

A limitation of this study is the timing of checking the blood cell ratios. The impact of neoadjuvant chemotherapy on blood cell ratios is not extensively analyzed yet. In a study, Li et al. discovered a significant decrease for NLR, PLR, LMR, and SII but no significant decrease for PNI after neoadjuvant chemotherapy for gastric cancer patients [95]. Liu et al. discovered an decrease in 75 of 111 patients as well as an increase in 36 of 111 patients in NLR for advanced gastric cancer treated with neoadjuvant chemotherapy [96], thus further research is needed on this topic. To minimize this bias in our study, we attempted to include studies with measurements before treatment (before surgery when just surgery was performed or before the neoadjuvant treatment when this was the first performed treatment). Most of the studies excluded patients’ treatment with neoadjuvant chemotherapy, and just 12 studies were enrolled which used neoadjuvant treatment. In seven studies the blood was taken before surgery, otherwise before neoadjuvant chemotherapy. Just seven studies took a blood sample after neoadjuvant chemotherapy. As the patients’ number was very low in comparison to the cohort (6x <5%, 1x 10%, and 1x 13.9% of the enrolled patients), we see this as a low bias.

The variety of the distance to the treatment was more complex and was between “day before surgery” up to “2 months before surgery” or “time of diagnosis”. In 34 of the 65 studies, the patients’ blood was taken 1–2 weeks before treatment. However, we did not find any study that addressed this topic so further studies should measure at different time points so an optimal time point could be evaluated in the future.

Another topic that should be addressed in further research is the role of blood cell ratios as a predictor of postoperative complications. Radulescu et al. discovered a significant difference in developing fistulas and complications leading to death in patients with an increased NLR [97]. A recent performed meta-analysis showed a significant impact of postoperative complications on overall survival [98].

In general, the existing literature fails to provide robust evidence for a prognostic role of blood cell ratios in patients with gastric cancer undergoing surgery, while the abundance of the available data indicates a potential relevance. The main reason for the current dilemma is the lack of evaluation of the same cut-off value for each ratio. Almost all studies are providing training sets defining a cut-off value without providing a validation set. Even more troublesome, many studies define new cut-off values, which makes it difficult to compare the studies amongst each other. Redundantly defining a new cut-off value hampers the idea of finding a relevant general cut-off value. For the definition of a clear biomarker, robust reproducible results are of highest importance.

It should be emphasized that the quantitative results in this article need to be interpreted with caution as several of the meta-regressions are based upon few studies only. This may be a possible reason as to why there was a significant association for LMR with 5-year survival, however not with OS and why there was a significant association for PNI with OS but not with 5-year survival. Another possible reason is, again, the difference of the used cut-offs.

Moreover, the quality of the available studies was moderate to low. Meta-regressions made cannot be considered as reliable as the results from SROC analyses or larger meta-regressions, as overfitting might have occurred in some cases, which might have led to spurious findings.

As seen in our systematic review, the most common used blood cell ratios with the highest significant associations are NLR with 27 (30) studies = 90% significant association, PLR 18 (19) studies = 94.7% significant association, and PNI 10 (12) studies = 83.3% significant association for overall survival, as well as NLR 9 (11) studies = 81.8% significant association for DFS and NLR 15 (18) studies = 83.3% significant association, and PNI 10/10 studies = 100% significant association for the 5-year survival rate. After our analysis, it is believed that NLR, PLR, and PNI are the blood cell ratios with the highest relevance in clinical settings to be used as a prognostic factor in patients with gastric cancer undergoing surgery.

## Figures and Tables

**Table 1:** Overview of investigated blood cell ratios.

Blood Cell Ratio	Calculation	Hypothesized Association
PLR	Platelets/lymphocytes	Low ratio with long survival
NLR	Neutrophiles/lymphocytes	Low ratio with long survival
MLR	Monocytes/lymphocytes	Low ratio with long survival
LMR	Lymphocytes/monocytes	High ratio with long survival
PNI	(10 × albumin) + (0.005 × lymphocytes)	High ratio with long survival
SII	Platelets × neutrophils/lymphocytes	Low ratio with long survival

Abbreviation: PNI: Prognostic nutritional index; LMR: Lymphocyte to monocyte ratio; SII: Systemic immune-inflammation index; MLR: Monocyte to lymphocyte ratio; NLR: Neutrophil to lymphocyte ratio; and PLR: Platelet to lymphocyte ratio.

**Table 2:** Overview of all used studies, investigated ratio, its cut-off and the publication bias assessed by the Quality in Prognosis Studies (QUIPS) tool. **OR = odds ratio, OS = overall survival, DFS = disease-free survival**.

Study	Patients	Survival Type	Study Participation	Study Attrition	Prognostic Factor Measurement	Outcome Measurement	Study Confounding	Statistical Analysis and Reporting	Investigated Blood Cell Ratio (Cut-Off)
Aurello 2014 [22]	102	OS, DFS	low	moderate	moderate	moderate	low	moderate	PNI (45), NLR (5)
Eo 2015 [23]	314	OS, DFS, OR	low	high	moderate	moderate	moderate	low	PNI (47.3)
Fujiwara 2016 [24]	62	OS	moderate	high	moderate	moderate	high	moderate	PNI (48)
Ishizuka 2014 [25]	154	OS	moderate	high	moderate	moderate	high	moderate	PNI (45), NLR (2.6)
Lee 2016 [26]	7781	OS, DFS	low	moderate	moderate	low	moderate	low	PNI (46.7), NLR (2.43)
Lin 2019 [27]	2182	OS	low	moderate	moderate	low	high	low	PNI (46.7)
Liu 2017 [28]	1330	OS	low	moderate	low	low	moderate	low	PNI (45)
Luo 2019 [29]	128	OS, DFS	low	high	high	moderate	low	low	PNI (50)
Migita 2013 [30]	548	OS, OR	low	high	moderate	moderate	high	low	PNI (48)
Sakurai, K. 2015 [31]	594	OS	moderate	high	moderate	moderate	high	low	PNI (45)
Sun 2017 [32]	117	OS, OR	moderate	moderate	moderate	low	low	moderate	PNI (45)
Jiang 2014 [33]	377	OR	low	moderate	moderate	moderate	low	moderate	PNI (46)
Murakami 2017 [34]	254	OR	moderate	high	moderate	moderate	high	moderate	PNI (52)
Nozoe 2009 [35]	248	OR	low	moderate	moderate	high	high	high	PNI (49.7)
Pan 2015 [36]	207	OR	moderate	low	moderate	low	moderate	moderate	PNI (45), NLR (4)
Saito 2017 [37]	453	OR	moderate	high	moderate	moderate	high	moderate	PNI (46,7)
Song 2018 [38]	1150	OR	unclear	unclear	unclear	unclear	unclear	unclear	PNI (51.81)
Sun 2015 [39]	632	OR	moderate	moderate	moderate	moderate	moderate	high	PNI (48.2)
Zhang 2020 [40]	273	OS, DFS	moderate	high	moderate	moderate	moderate	moderate	PNI (41.25), NLR (3.32), PLR (185.8)
Hsu 2016 [41]	926	OS, OR	moderate	moderate	moderate	moderate	high	moderate	LMR (4.8)
Lin 2017 [42]	452	OS, DFS	low	moderate	moderate	low	moderate	low	LMR (3.15)
Lin 2018 [43]	1786	OS	low	moderate	moderate	low	moderate	low	LMR (3.4), NLR (4), PLR (161.3)
Pan 2018 [44]	870	OS, OR	moderate	moderate	moderate	low	low	moderate	LMR (5.43), NLR (1.44), PLR (115)
Lieto 2017 [45]	297	DFS, OR	low	moderate	moderate	low	low	moderate	LMR (3.37), NLR (3.22)
Cheng 2020 [46]	607	OS	moderate	moderate	moderate	moderate	high	moderate	LMR (3.91), NLR (3.41), PLR (141.3)
Xu 2020 [47]	401	OS, DFS	low	moderate	moderate	low	moderate	moderate	LMR (3.15)
Chen 2017 [48]	292	OS, DFS	low	moderate	moderate	low	moderate	moderate	SII (600), MLR (0.17), NLR (1.65), PLR (133)
Guo 2018 [49]	1058	OS	low	moderate	high	moderate	moderate	moderate	SII (521.6), NLR (2.5), PLR (152)
Liu 2017 [50]	1056	OS	low	moderate	moderate	moderate	moderate	low	NLR (2), PLR (130)
Shi 2018 [51]	688	OS	low	moderate	moderate	moderate	moderate	low	SII (320), MLR (0.23), NLR (1.30), PLR (135)
Wang 2017 [52]	444	OS	moderate	moderate	moderate	moderate	moderate	moderate	SII (660), NLR (2.1), PLR (120)
Lu 2018 [53]	401	DFS	low	moderate	moderate	low	unclear	low	SII (784.7), NLR (3.1), PLR (133.2)
Hirahara 2020 [54]	412	OS	low	moderate	moderate	moderate	moderate	moderate	SII (661.9), NLR (2.529), PLR (212.1)
Lin 2020 [55]	2257	OS	moderate	moderate	moderate	moderate	high	moderate	SII (569.93)
Chen 2017 [56]	91	OS, DFS	moderate	moderate	moderate	low	low	moderate	MLR (0,27)
Feng 2017 [57]	1621	OS	moderate	moderate	moderate	moderate	moderate	moderate	MLR (0.19), NLR (2,6), PLR (130.7)
Li 2017 [58]	455	DFS	low	moderate	moderate	moderate	moderate	moderate	MLR (0.22), NLR (2.10)
Chen 2017 [59]	91	OS, DFS	low	moderate	moderate	low	moderate	moderate	NLR (2.17)
Ghidini 2019 [60]	186	OS	moderate	high	high	moderate	high	moderate	NLR (2.54)
Gong 2017 [61]	91	OS	low	moderate	moderate	high	moderate	moderate	NLR (1.44), PLR (161)
Kim 2015 [62]	1986	OS	low	high	moderate	moderate	moderate	moderate	NLR (1.99), PLR (126)
Lian 2015 [63]	162	OS, DFS	low	high	low	moderate	high	moderate	NLR (4.02), PLR (208)
Lin 2019 [64]	1167	OS	low	low	moderate	moderate	moderate	low	NLR (2.6), PLR (160.7)
Min 2017 [65]	734	OS, OR	low	high	moderate	moderate	high	low	NLR (3)
Mohri 2010 [66]	357	OS, OR	low	high	low	moderate	low	moderate	NLR (2.2)
Mohri 2016 [67]	404	OS	moderate	moderate	moderate	low	high	moderate	NLR (3)
Shimada 2010 [68]	1028	OS, OR	moderate	moderate	moderate	high	high	moderate	NLR (4)
Sun 2016 [69]	873	OS	low	moderate	moderate	moderate	moderate	moderate	NLR (2.3), PLR (117)
Szor 2018 [70]	383	OS, DFS	low	high	moderate	high	moderate	moderate	NLR (2,44)
Ubukata 2010 [71]	157	OS, OR	low	high	moderate	moderate	moderate	moderate	NLR (5)
Yamamoto 2019 [72]	666	OS, DFS, OR	moderate	high	moderate	moderate	high	moderate	NLR (2,5)
Zhang 2018 [73]	904	OS, OR	moderate	moderate	moderate	high	high	moderate	NLR (2), PLR (160)
Zhou 2018 [74]	103	OS, DFS	moderate	moderate	moderate	moderate	moderate	moderate	NLR (2,76)
Zhou 2016 [75]	451	OS	moderate	high	moderate	high	moderate	moderate	NLR (2,76), PLR (167)
Fang 2017 [76]	190	OR	high	low	moderate	low	moderate	high	NLR (2)
Graziosi 2015 [77]	156	OR	low	moderate	moderate	low	high	high	NLR (2.34)
Hsu 2015 [78]	1030	OR	moderate	moderate	moderate	moderate	high	moderate	NLR (3.44), PLR (132)
Jiang 2014 [79]	377	OR	low	moderate	moderate	moderate	low	moderate	NLR (1.44), PLR (184)
Lee 2013 [80]	220	OR	moderate	high	moderate	high	high	high	NLR (2.15)
Miyatani 2017 [81]	280	OR	moderate	high	high	high	high	moderate	NLR (2,7)
Qiu 2015 [82]	706	OR	moderate	high	high	moderate	moderate	moderate	NLR (3)
Saito 2017 [83]	453	OR	moderate	high	moderate	moderate	high	moderate	NLR (2.43)
Yu 2015 [84]	291	OR	low	moderate	moderate	moderate	moderate	moderate	NLR (3,5)
Liu 2015 [85]	455	OS	low	high	moderate	moderate	high	moderate	SII (660), PLR (180)
Saito 2018 [86]	453	OR	moderate	high	moderate	moderate	high	moderate	PLR (173.3)

**Table 3:** Summary of quantitative results showing significant studies, used cut-offs, the results for meta-regression for the cut-offs as the optimal cut-off analyzed by SROC.

	OS	DFS	5-Year Survival Rate
**PNI**			
Significant studies	10/12 (83%)	**5/5 (100%)**	**10/10 (100%)**
Used cut-offs	**50, 48 (1 sign.,** 1 not sign.), **47.3, 46.7, 46, 45 (4 sign.,** 1 not sign.), **41.25**	**50, 47.3, 46.7, 45, 41.25**	**52, 51.81, 49.7, 48.2, 47.3, 46.7, 46, 45 (2x)**
Meta-regression for cut-off	***p* = 0.026**	***p* = 0.032**	*p* = 0.284
SROC		not possible	
Suggested cut-off based on this analysis		45	
**LMR**			
Significant studies/ total studies (%)	**6/6 (100%)**	**3/3 (100%)**	**3/3 (100%)**
Used cut-offs	**5.43, 4.8, 3.91, 3.4, 3.15 (2x)**	**3.37, 3.15 (2x)**	**5.43, 4.8, 3.37**
Meta-regression for cut-off	*p* = 0.253	*p* = 0.853	***p* = 0.020**
SROC		not possible	
Suggested cut-off based on this analysis		5.43	
**SII**			
Significant studies/ total studies (%)	**7/7 (100%)**	**2/2 (100%)**	n/a
Used cut-offs	**320, 521.6, 569.93, 600, 660 (2x), 661.9,**	**600, 784.7**	n/a
Meta-regression for cut-off	*p* = 0.171	n/a	n/a
SROC		not possible	
Suggested cut-off based on this analysis		320	
**MLR**			
Significant studies/ total studies (%)	2/4 (50%)	1/3 (33%)	n/a
Used cut-offs	0.17, **0.19, 0.23**, 0.27	0.17, **0.22, 0.27**	**n/a**
Meta-regression for cut-off	*p* = 0.695	*p* = 0.660	n/a
SROC		not possible	
Suggested cut-off based on this analysis		0.9	
**NLR**			
Significant studies/ total studies (%)	27/30 (90%)	9/11 (82%)	15/18 (83%)
Used cut-offs	**1.3, 1.44 (1 sign.,** 1 not sign.), 1.65, **1.99, 2 (2x), 2.1**, 2.17, **2.2, 2.3, 2.44, 2.5 (2x), 2.529, 2.54, 2.6 (3x), 2.76, 3 (2x), 3.32, 4 (2x), 4.02, 5 (2x)**	1.65, **2.1,** 2.17, **2.44, 2.5, 2.76, 3.1, 3.22, 3.32, 4.02, 5**	**1.44 (2x), 2 (1 sign,** 1 not sign.), 2.15, **2.2, 2.34, 2.43, 2.5, 2.7, 3 (1 sign,** 1 not sign,), **3.22, 3.44, 3.5, 4, 5**
Meta-regression for cut-off	***p* = 0.002**	***p* = 0.006**	***p* = 0.001**
SROC	Optimal cut-off of 4.506		
Suggested cut-off based on this analysis		4.5	
**PLR**			
Significant studies/ total studies (%)	18/19 (94%)	3/5 (60%)	**4/4 (100%)**
Used cut-offs	**115, 117, 120, 126, 130, 130.7,** 133, **135, 141.3, 152, 160, 160.7, 161, 161.3 167, 180, 185.8, 208, 212,1**	**126,** 133, 133.2, **185.8, 208**	**115, 132, 173.3, 184**
Meta-regression for cut-off	*p* = 0.144	*p* = 0.659	*p* = 0.629
SROC	Optimal cut-off of 152.47		
Suggested cut-off based on this analysis		152	

Bold writing: ≥95% significant studies, *p* < 0.05 or SROC available. Abbreviations: n/a: not applicable; SROC: summary receiver operating characteristic.

**Table 4:** Recommendations for optimal cut-off values for future studies and its grade of recommendation.

Blood Cell Ratio	Recommended Cut-Off	Grade of Recommendation
PNI	45	HIGH
LMR	5.43	MODERATE
SII	320	MODERATE
MLR	0.19	LOW
NLR	4.5	HIGH
PLR	152	HIGH

Abbreviation: PNI: Prognostic nutritional index; LMR: Lymphocyte to monocyte ratio; SII: Systemic immune-inflammation index; MLR: Monocyte to lymphocyte ratio; NLR: Neutrophil to lymphocyte ratio, PLR: Platelet to lymphocyte ratio.

## Data Availability

Extracted Data are available upon request.

## Supplementary Materials

The following supporting information can be downloaded at: www.mdpi.com/article/10.3390/diagnostics12030593/s1, Table 3 Figure S1: Funnel plot for (A) OS for PNI, (B) OS for NLR, (C) DFS for NLR, and (D) 5-year survival rate for NLR, Figure S2: Meta regression for (A) OS for PNI, (B) DFS for PNI, (C) 5-year survival rate for PNI, (D) OS for LMR, (E) DFS for LMR, and (F) 5-year survival rate for LMR, Figure S3: Forest plot for (A) DFS for PNI, (B) 5-year survival rate for PNI, (C) DFS for LMR, (D) 5-year survival rate for LMR, (E) DFS for SII, and (F) DFS for MLR, Figure S4: Meta regression for (A) OS for SII, (B) OS for MLR, (C) DFS for MLR, (D) OS for NLR, (E) DFS for NLR, and (F) 5-year survival rate for NLR, Figure S5: SROC for (A) NLR, (B) PLR, Figure S6: Forest plot for (A) DFS for NLR, (B) 5-year survival rate for NLR, (C) DFS for PLR, and (D) 5-year survival rate for PLR, Figure S7: Meta regression for (A) OS for PLR, (B) DFS for PLR, and (C) 5-year survival rate for PLR.
