# Supplementary figures and images for: Systematic Review of Prognostic Role of Blood Cell Ratios in Patients with Gastric Cancer Undergoing Surgery
Source: Diagnostics (Basel). 2022 Feb 25;12(3):593. doi: 10.3390/diagnostics12030593 (PMC8947199; doi:10.3390/diagnostics12030593)

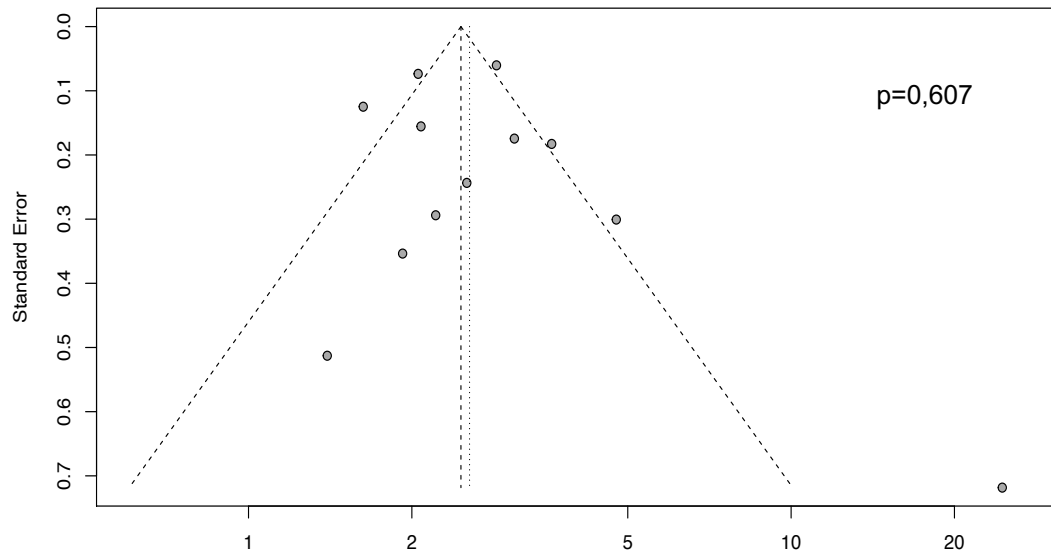

A

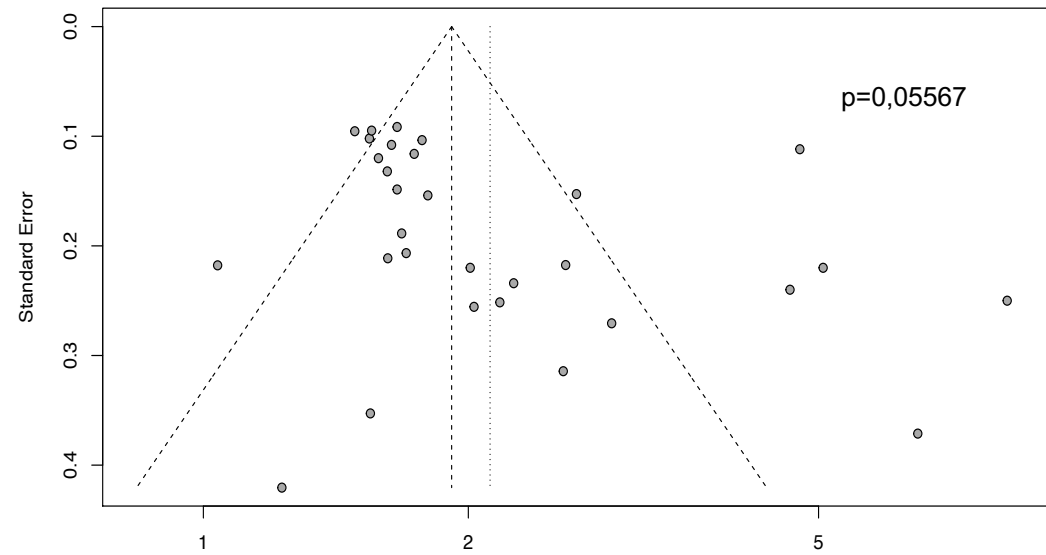

B

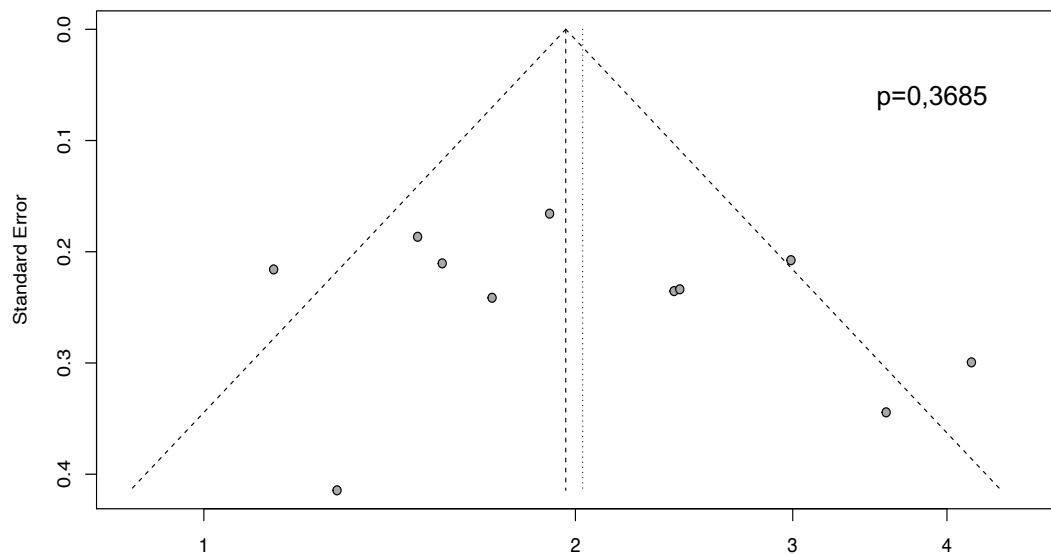

C

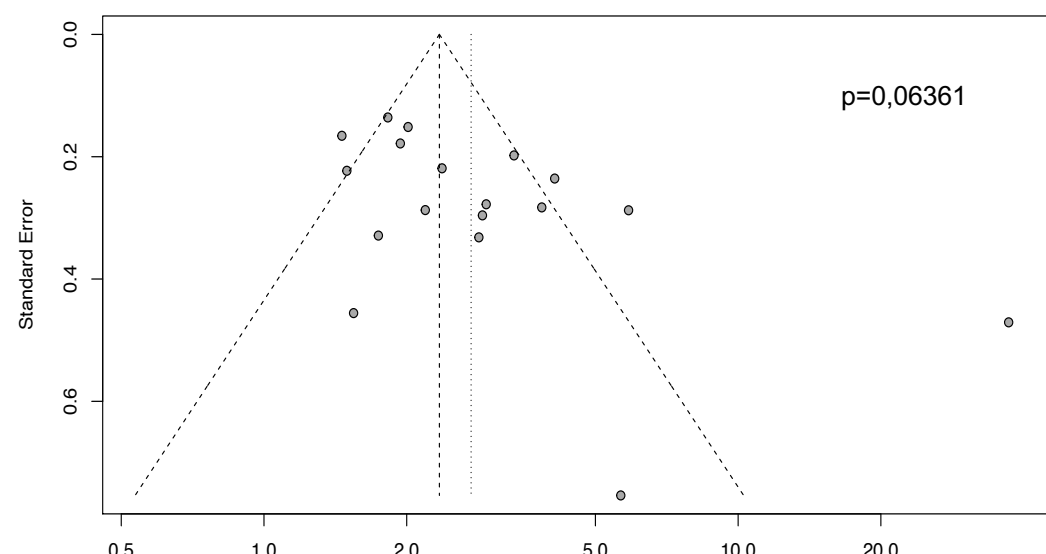

D

Supplement: Supplementary file 1 [file diagnostics-12-00593-s001.zip › Figure S1A-D.pdf]

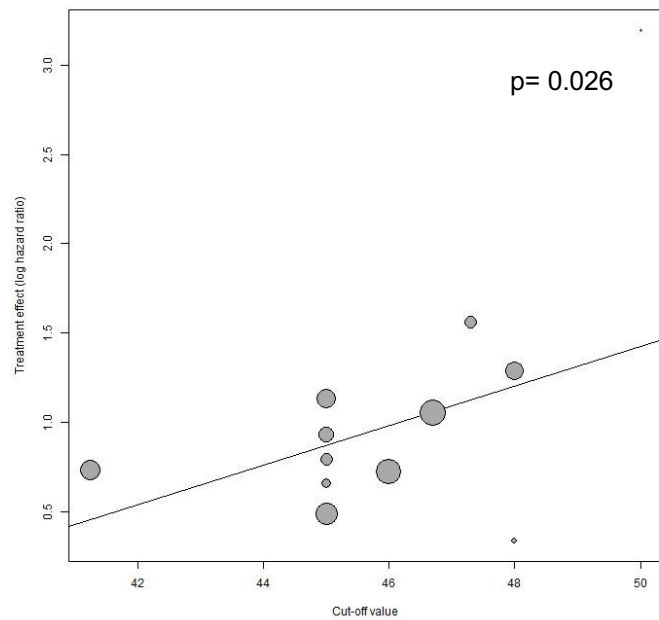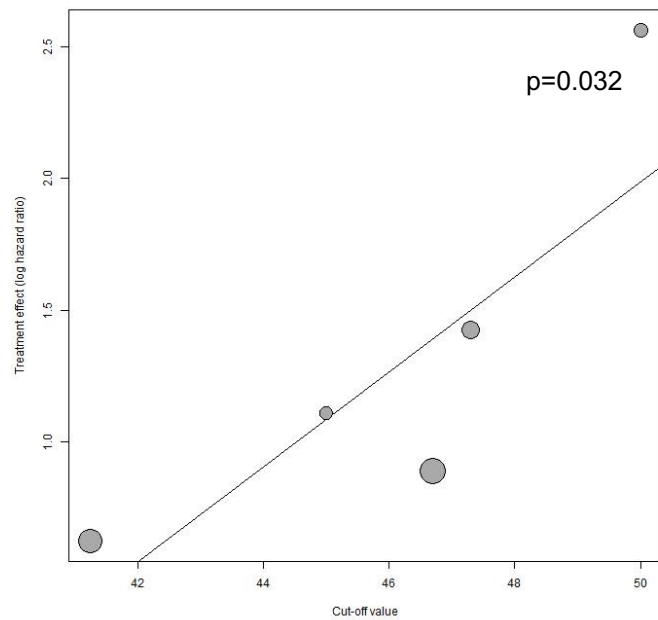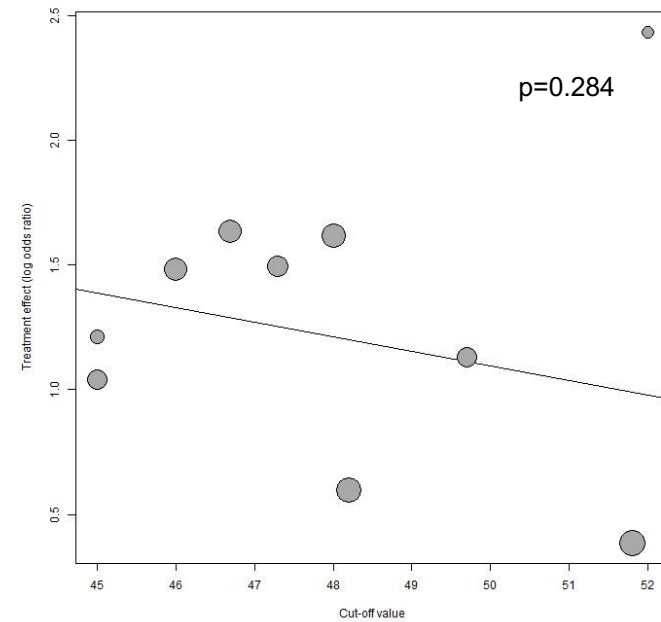

A

B

C

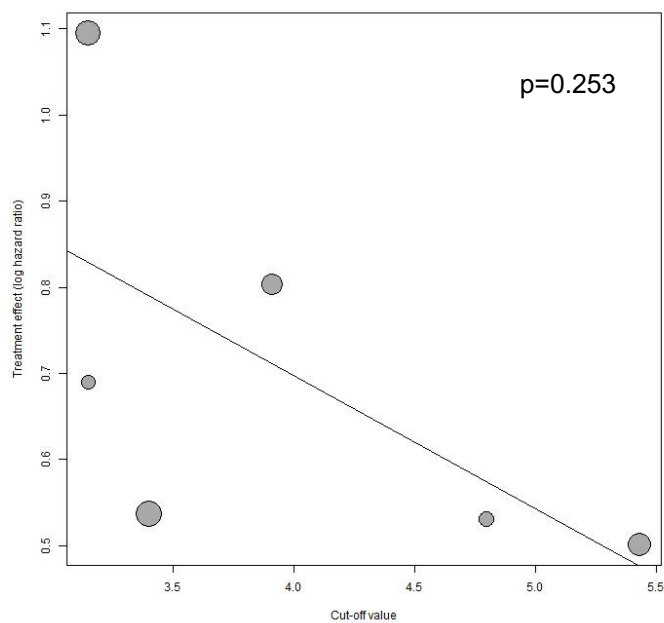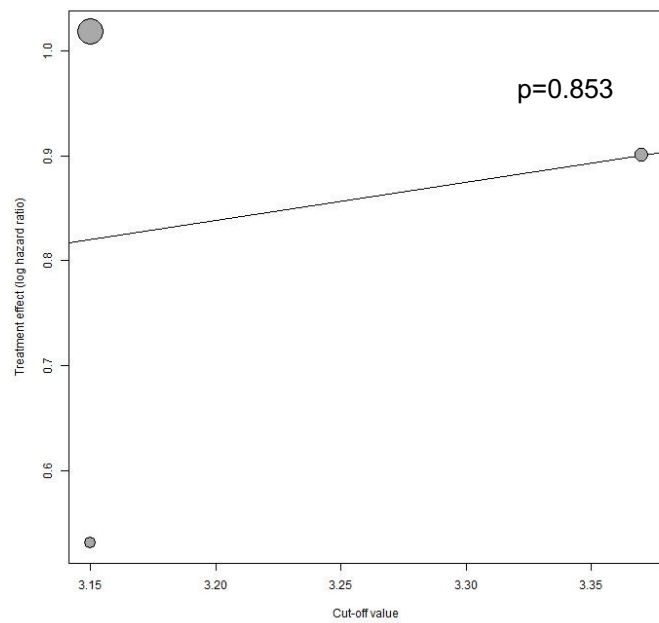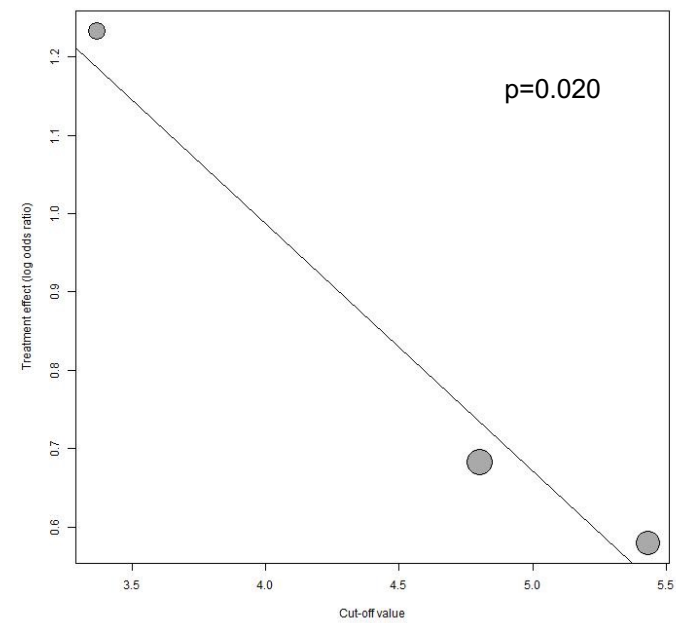

D

E

F

Supplement: Supplementary file 1 [file diagnostics-12-00593-s001.zip › Figure S2A-F.pdf]

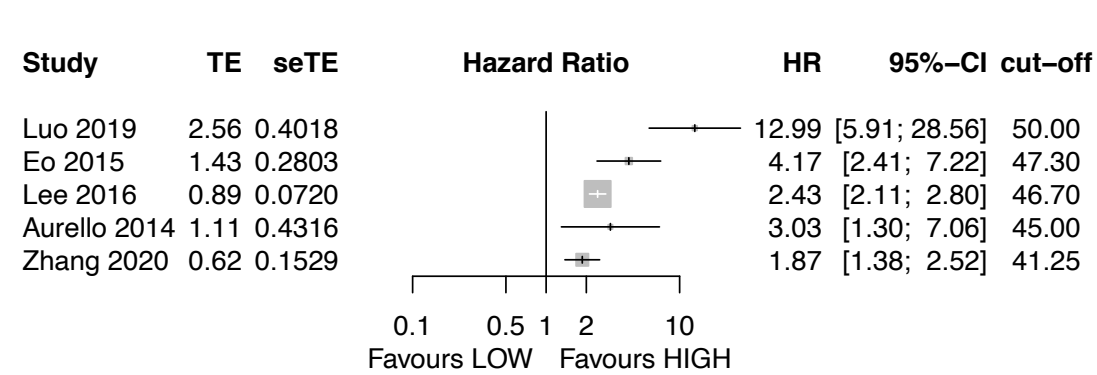

A

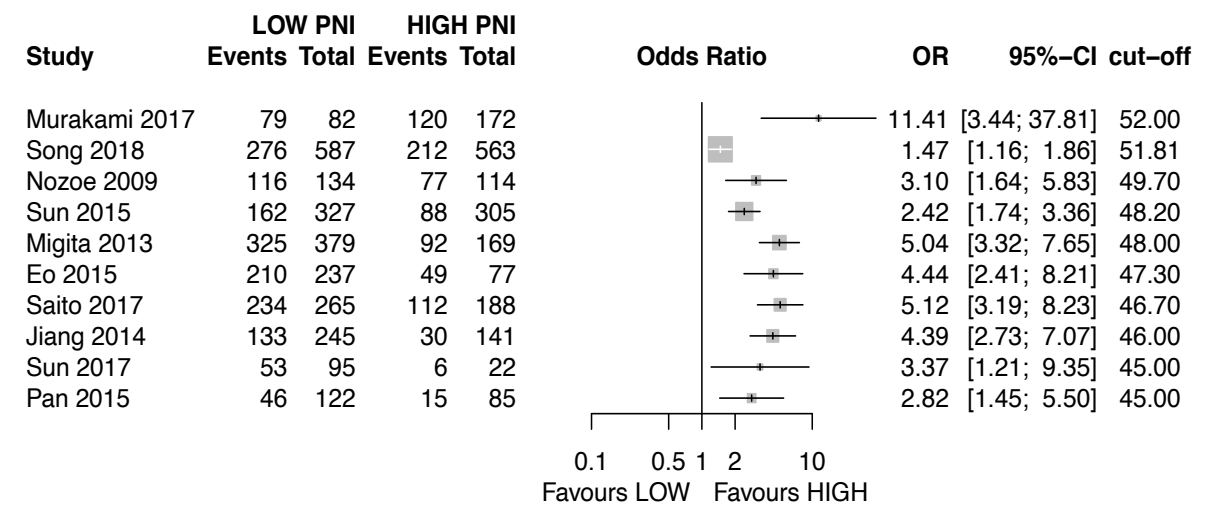

B

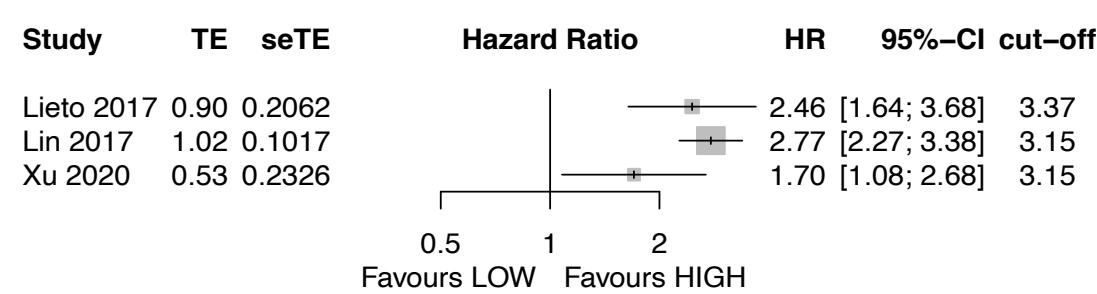

C

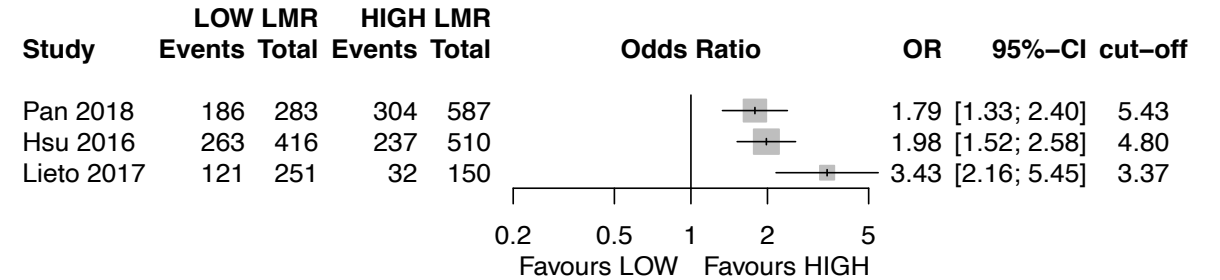

D

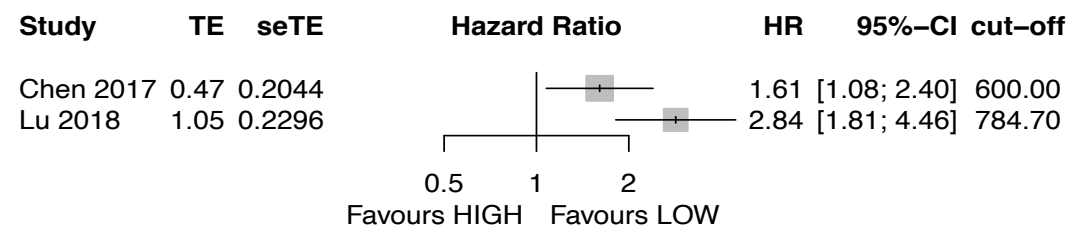

E

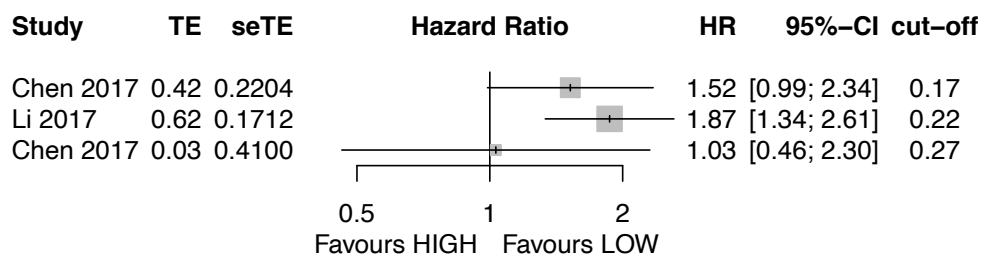

F

Supplement: Supplementary file 1 [file diagnostics-12-00593-s001.zip › Figure S3A-F.pdf]

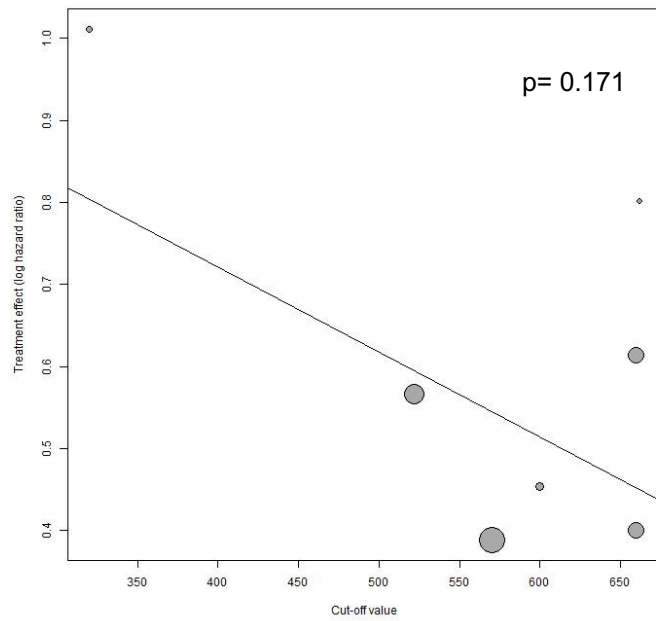

A

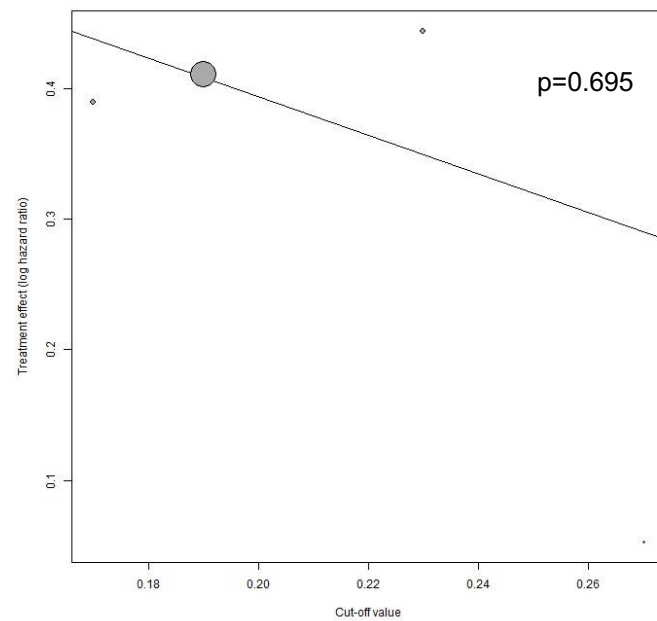

B

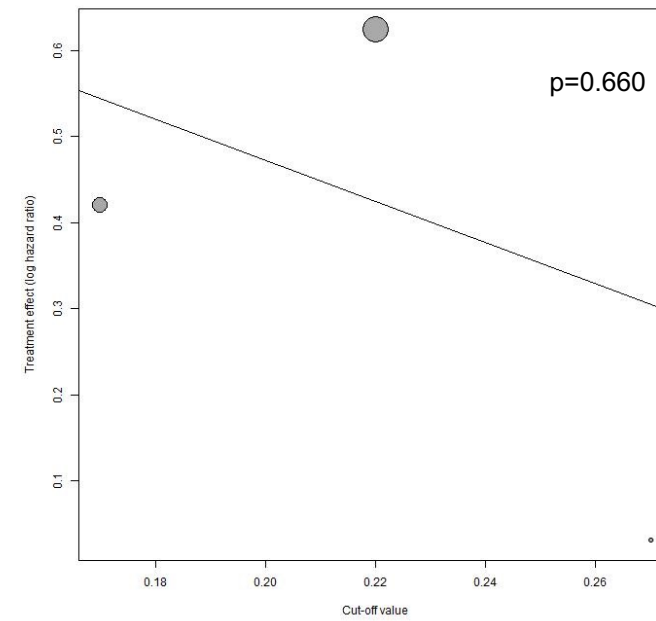

C

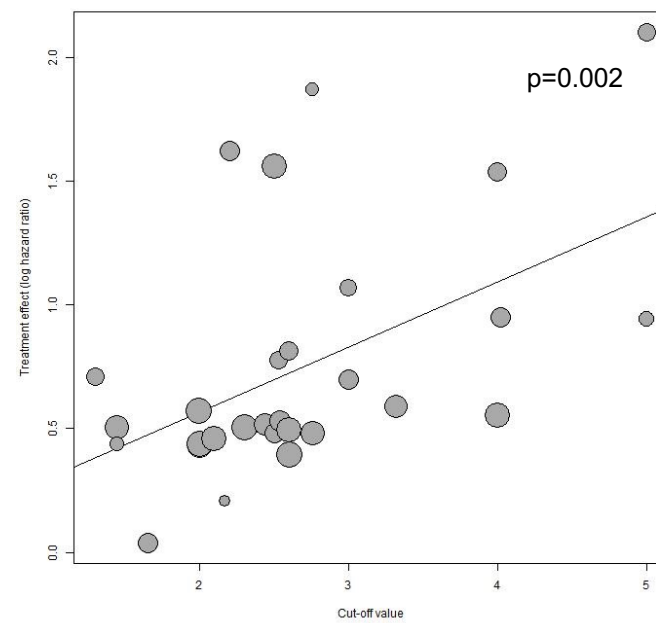

D

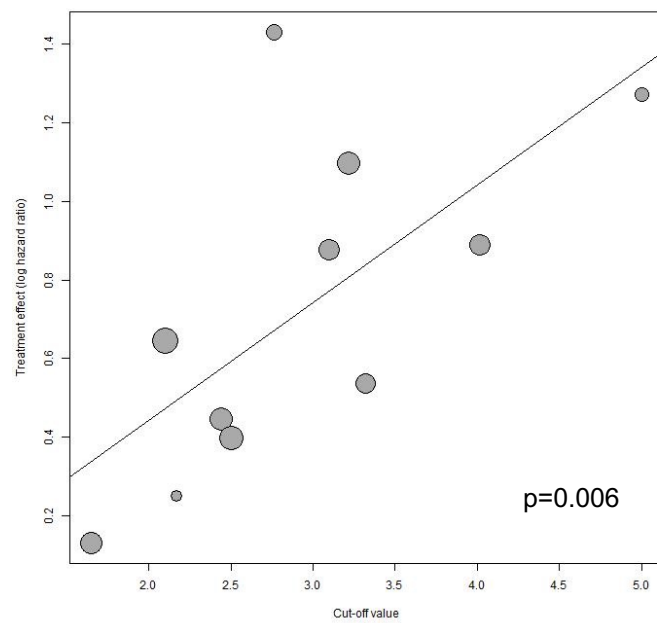

E

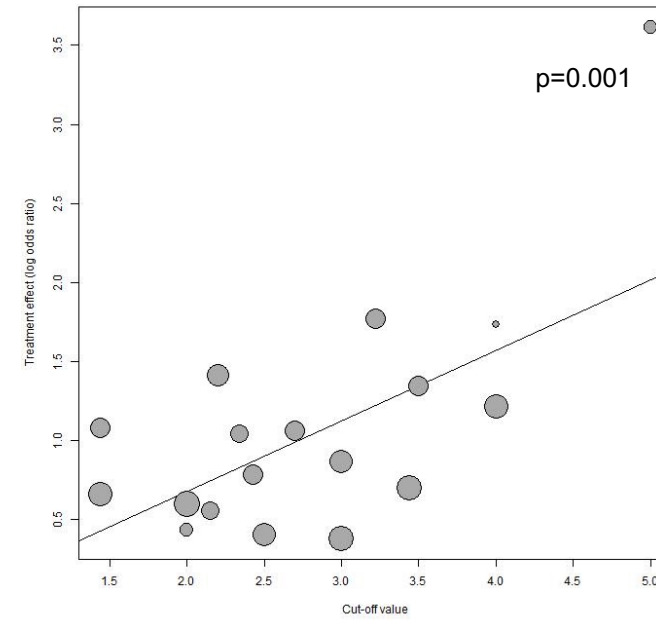

F

Supplement: Supplementary file 1 [file diagnostics-12-00593-s001.zip › Figure S4A-F.pdf]

SROC curve

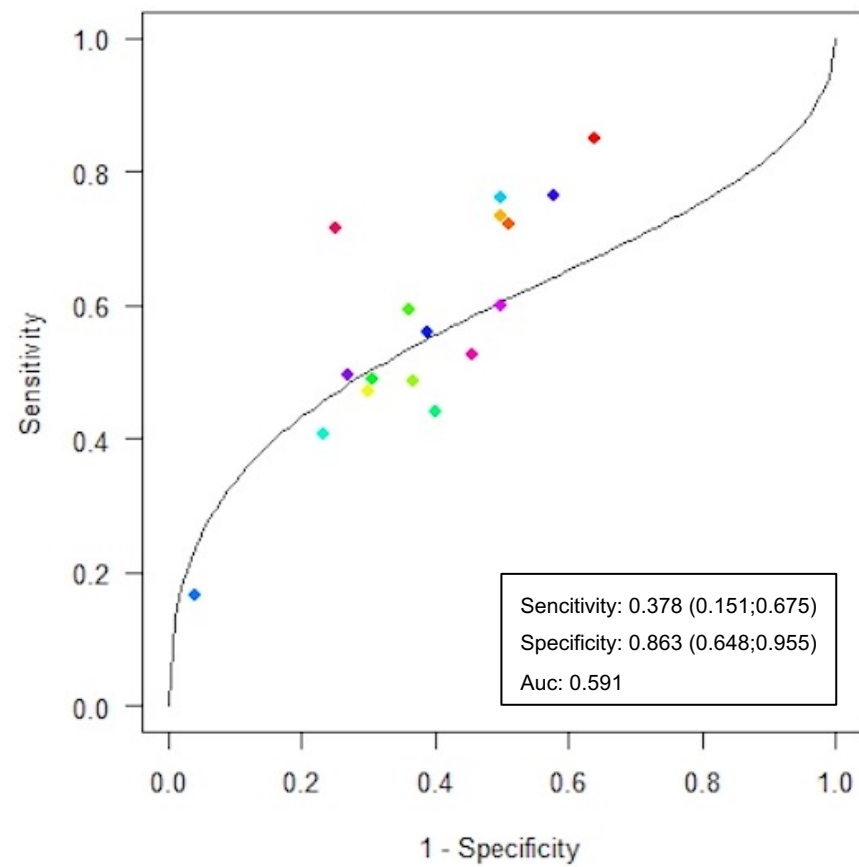

A

SROC curve

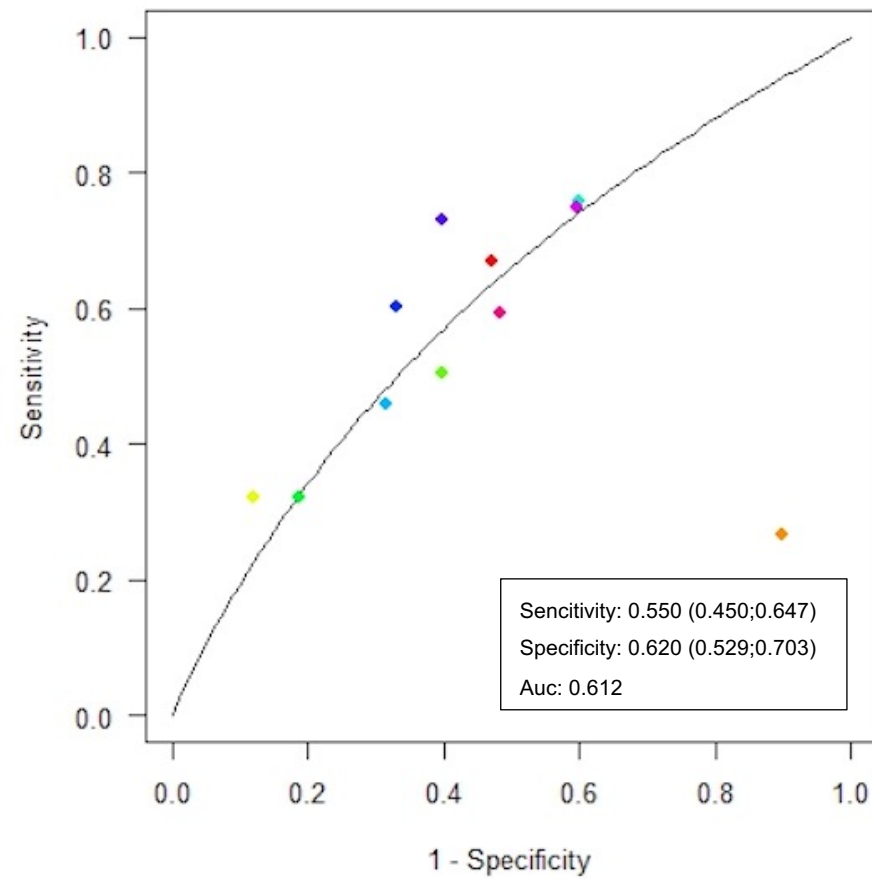

B

Supplement: Supplementary file 1 [file diagnostics-12-00593-s001.zip › Figure S5A+B.pdf]

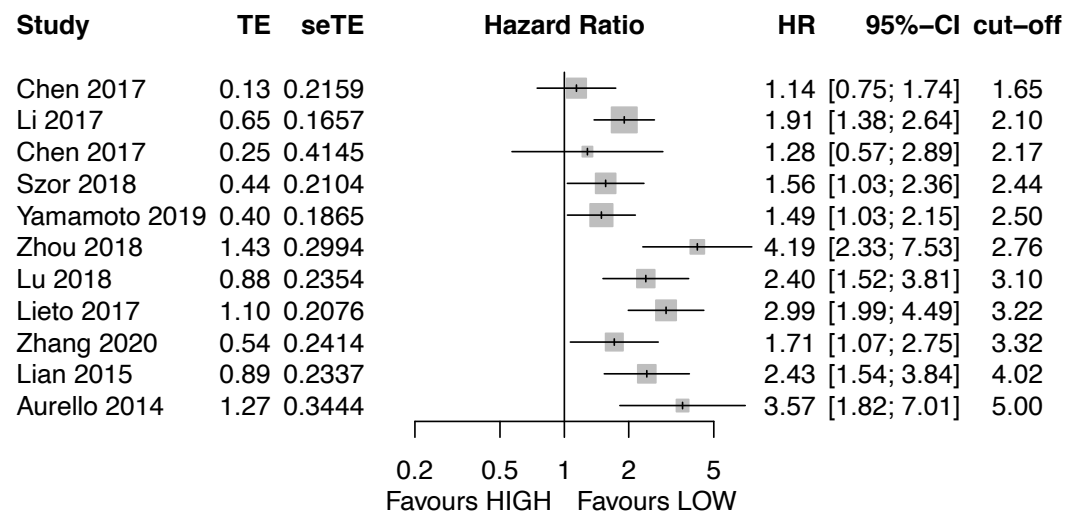

A

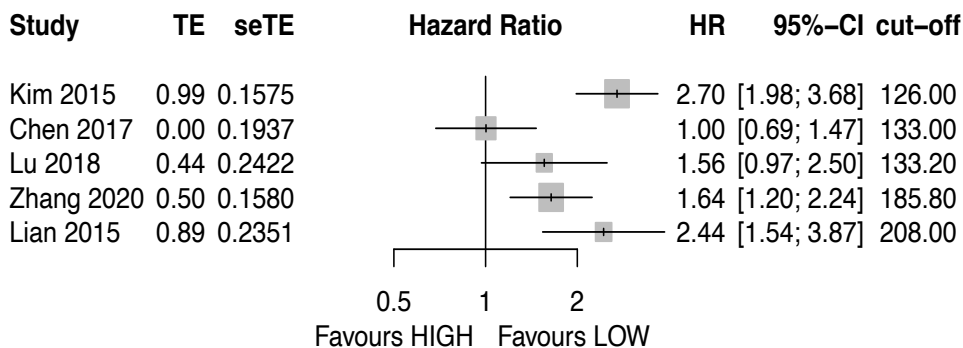

C

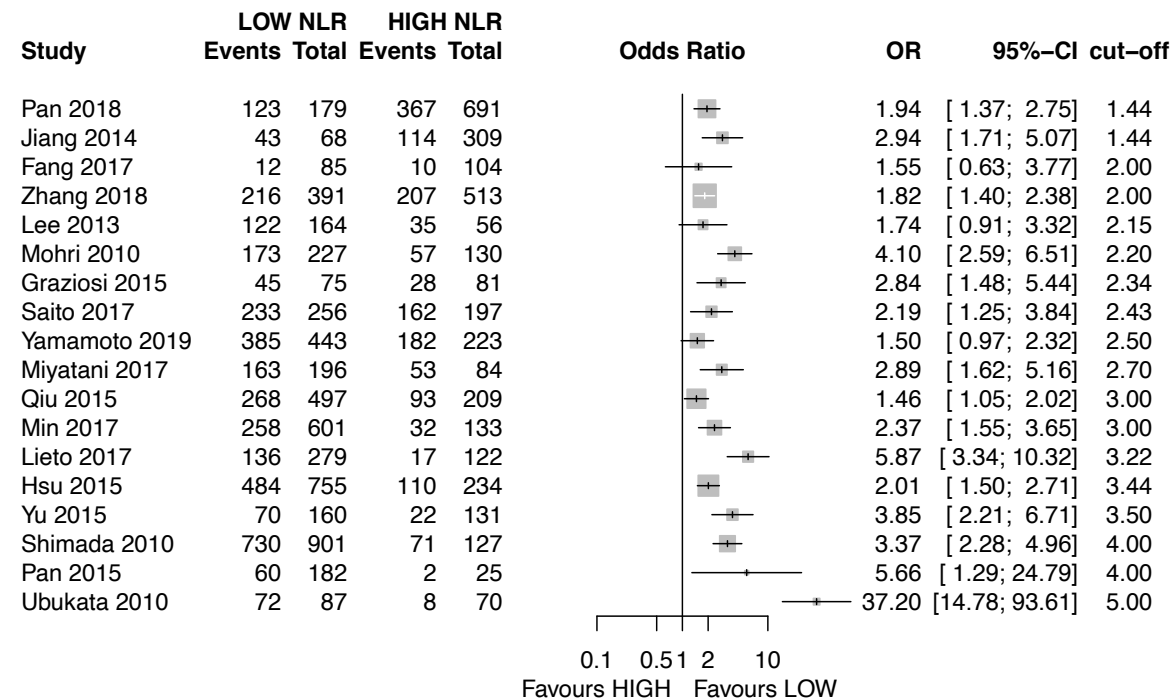

B

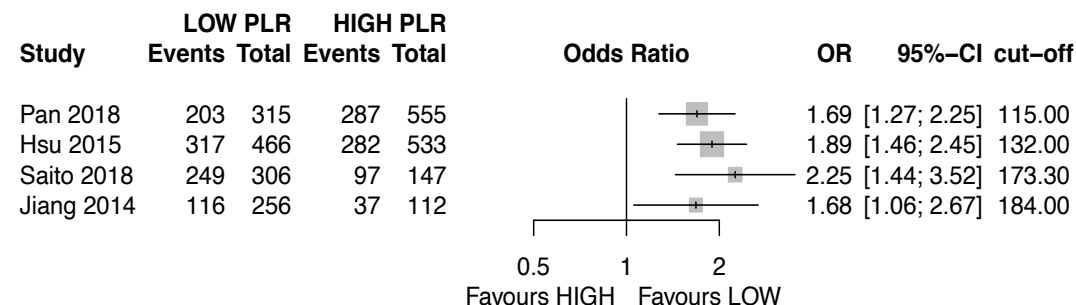

D

Supplement: Supplementary file 1 [file diagnostics-12-00593-s001.zip › Figure S6A-D.pdf]

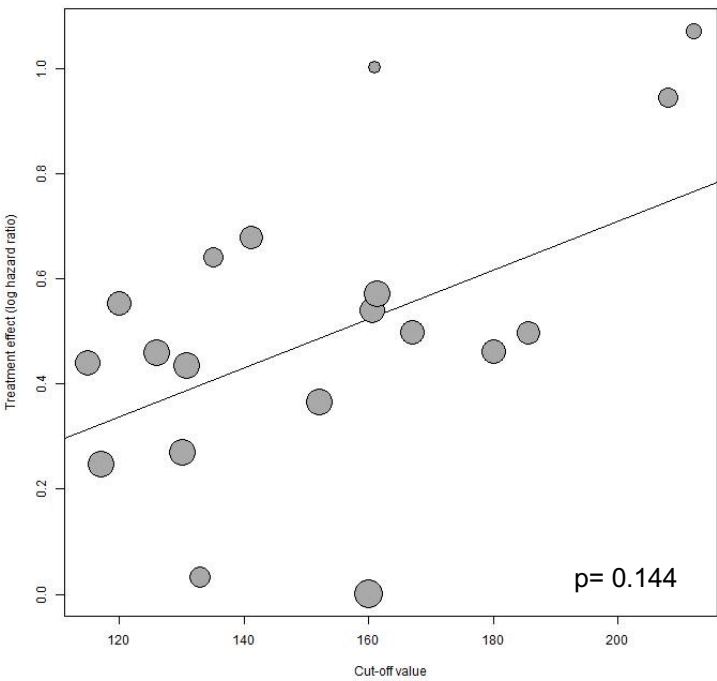

A

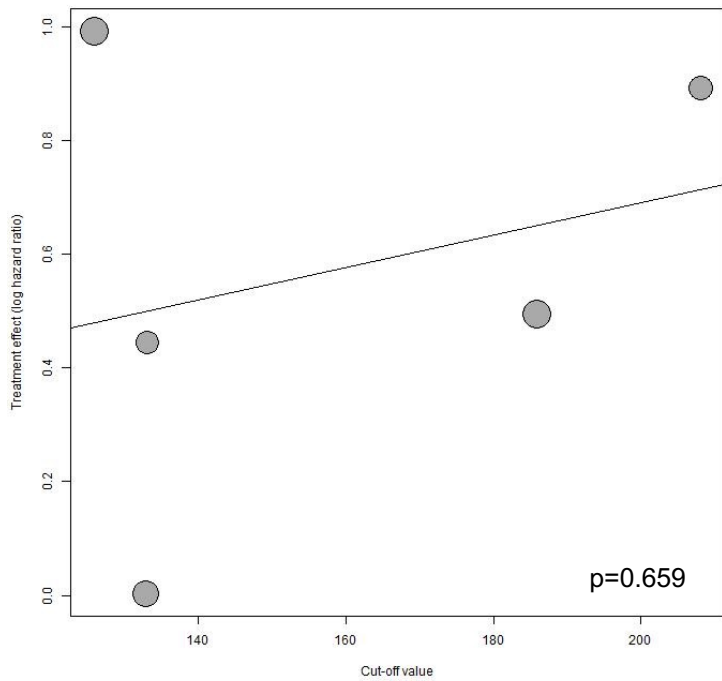

B

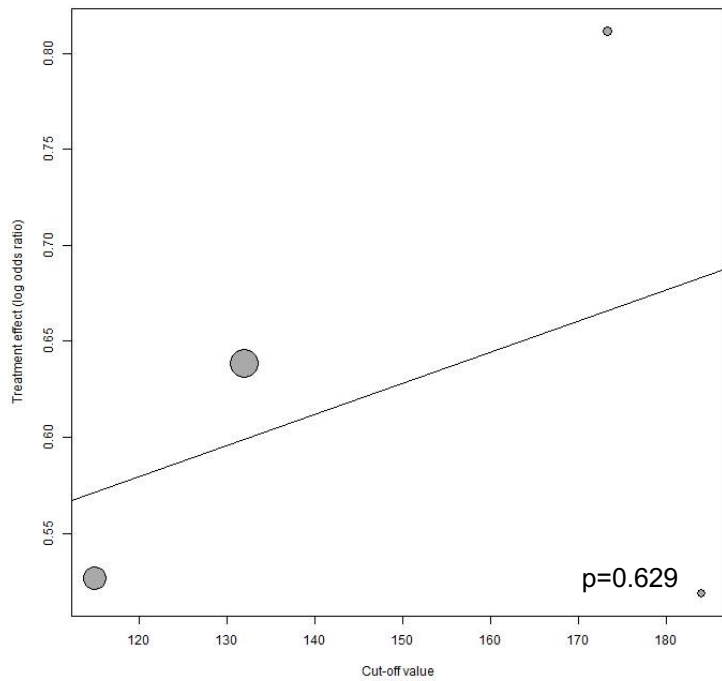

C

Supplement: Supplementary file 1 [file diagnostics-12-00593-s001.zip › Figure S7A-C.pdf]
